# Regulation of biotic interactions and responses to abiotic stresses by MAP kinase pathways in plant pathogenic fungi

**DOI:** 10.1007/s44154-021-00004-3

**Published:** 2021-08-18

**Authors:** Xue Zhang, Zeyi Wang, Cong Jiang, Jin-Rong Xu

**Affiliations:** 1 State Key Laboratory of Crop Stress Biology for Arid Areas and NWAFU-Purdue Joint Research Center, College of Plant Protection, Northwest A&F University, Yangling, 712100 Shaanxi China; 2 Department of Botany and Plant Pathology, Purdue University, West Lafayette, IN 47907 USA

**Keywords:** Fungal pathogens, Signal transduction, Pathogenesis, Virulence, Oxidative stress, Osmotic stress, Fungicide resistance, Bacterial-fungal interactions

## Abstract

Like other eukaryotes, fungi use MAP kinase (MAPK) pathways to mediate cellular changes responding to external stimuli. In the past two decades, three well-conserved MAP kinase pathways have been characterized in various plant pathogenic fungi for regulating responses and adaptations to a variety of biotic and abiotic stresses encountered during plant infection or survival in nature. The invasive growth (IG) pathway is homologous to the yeast pheromone response and filamentation pathways. In plant pathogens, the IG pathway often is essential for pathogenesis by regulating infection-related morphogenesis, such as appressorium formation, penetration, and invasive growth. The cell wall integrity (CWI) pathway also is important for plant infection although the infection processes it regulates vary among fungal pathogens. Besides its universal function in cell wall integrity, it often plays a minor role in responses to oxidative and cell wall stresses. Both the IG and CWI pathways are involved in regulating known virulence factors as well as effector genes during plant infection and mediating defenses against mycoviruses, bacteria, and other fungi. In contrast, the high osmolarity growth (HOG) pathway is dispensable for virulence in some fungi although it is essential for plant infection in others. It regulates osmoregulation in hyphae and is dispensable for appressorium turgor generation. The HOG pathway also plays a major role for responding to oxidative, heat, and other environmental stresses and is overstimulated by phenylpyrrole fungicides. Moreover, these three MAPK pathways crosstalk and coordinately regulate responses to various biotic and abiotic stresses. The IG and CWI pathways, particularly the latter, also are involved in responding to abiotic stresses to various degrees in different fungal pathogens, and the HOG pathway also plays a role in interactions with other microbes or fungi. Furthermore, some infection processes or stress responses are co-regulated by MAPK pathways with cAMP or Ca^2+^/CaM signaling. Overall, functions of individual MAP kinase pathways in pathogenesis and stress responses have been well characterized in a number of fungal pathogens, showing the conserved genetic elements with diverged functions, likely by rewiring transcriptional regulatory networks. In the near future, applications of genomics and proteomics approaches will likely lead to better understanding of crosstalk among the MAPKs and with other signaling pathways as well as roles of MAPKs in defense against other microbes (biotic interactions).

## Introduction

Plant pathogenic fungi are exposed to a variety of abiotic and biotic stresses in their natural habitats as well as during infectious growth, such as hyperosmolarity, extreme pH or temperature, mycoparasitic organisms, oxidative burst, and phytoalexins. Effective stress perception and responses are necessary for adaptation, survival, and overcoming plant defense. Like other eukaryotic organisms, fungi have the well-conserved mitogen-activated protein (MAP) kinase pathways that play important roles in responding to external stimuli (Chen and Thorner 2007; Brewster and Gustin 2014). In the past two decades, MAP kinase (MAPK) pathways have been functionally characterized in various plant pathogenic fungi that differ in dispersal, survival, host ranges, and infection mechanisms. In addition to their roles in infection and developmental processes, MAPKs in fungal pathogens are important for regulating responses to various environmental stresses and interactions with other microbes (Hamel et al. 2012; Braunsdorf et al. 2016; Jiang et al. 2018).

The typical MAP kinase pathway comprises a protein kinase cascade consisting of a MAP kinase (MAPK), a MAPK kinase (MEK), and a MEK kinase (MEKK). The sequential activation of these protein kinases in response to extracellular signals results in the activation of MAPKs, which then phosphorylate downstream target proteins and transcription factors (TFs) to regulate transcriptional and cellular changes. The budding yeast *Saccharomyces cerevisiae*, a model organism in which MAP kinase pathways are best characterized, have five MAPKs (Fus3, Kss1, Slt2, Hog1, and Smk1) that regulate pheromone response, filamentation/invasiveness, cell wall integrity, high osmolarity growth, and ascospore cell assembly (Schwartz and Madhani 2004; Chen and Thorner 2007). However, most plant pathogenic ascomycetes, the focus of this review, normally have only three MAPKs that are orthologous to yeast Fus3/Kss1, Slt2, and Hog1, and three corresponding upstream MEKs and MEKKs (Chen and Thorner 2007; Li et al. 2012), with only a few exceptions such as two HOG1 MAPKs in *Verticillium dahlia* and two BCK1 MEKKs in *Fusarium oxysporum*. These three well conserved MAPK pathways have both common and distinct biological functions in pathogenesis, differentiation, and stress responses in phytopathogenic fungi (Jiang et al. 2018). In this review, we will summarize general functions of fungal MAPKs in pathogenesis, but the emphasis will be on their roles in regulating responses to abiotic and biotic stresses.

## Roles of MAPK pathways in fungal pathogenesis

**Fig. 1:**
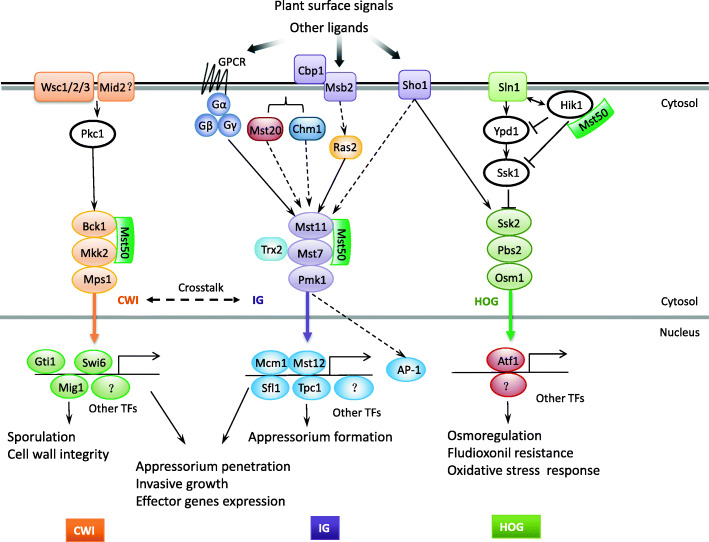
Functions of three MAPK pathways in the model plant pathogenic fungus *Magnaporthe oryzae.* Plant surface (chemical and physical) signals and other ligands of infected plant tissues are recognized by Sho1, two putative mucins (Msb2 and Cbp1), GPCR (such as Pth11), or other uncharacterized receptors to activate the downstream Pmk1 IG MAPK pathway possibly via Gα-GTP, freed Gβγ, PAK kinases (Mst20/Chm1), and Ras2. Mst50 is an adaptor protein that is also involved in the other two MAPK pathways. Thioredoxin Trx2 affects the activation of Mst7, which interacts with Pmk1 at the docking sites. Activated Pmk1 regulates the expression of genes important for appressorium formation, penetration, and invasive growth by Mst12, Mcm1, Sfl1, Tpc1, and possibly other transcription factors. (Mst12 interacts with Mcm1 and Tpc1, and Sfl1 directly interacts with Pmk1). For the Mps1 CWI pathway that functions downstream from PKC for regulating appressorium penetration, sporulation, and invasive growth, all the yeast cell wall integrity sensors are conserved in plant pathogenic fungi (Table 1). Transcription factors regulated by the Mps1 MAPK cascade include Mig1, Swi6, and Gti1, with the latter as a regulator of many effector genes. Both IG and CWI MAPK pathways are important for invasive growth and disease development. Unlike the other two MAPKs, the Osm1 MAPK is dispensable for plant infection although it has the conserved role in osmoregulation and sensitivity to fludioxonil. It is also important for responses to oxidative stress through transcription factor Atf1, which may be activated by other MAPKs or protein kinases because the *atf1* deletion mutant is defective in plant infection. Although Sho1 is an osmosensor in *M. oryzae*, it is also involved in activating the IG MAPK together with Msb2

Considering all the physical barriers and possible defense responses, the host plant is likely a ‘hostile’ habitat for fungal pathogens, which often use MAPK signaling to regulate infection-related morphogenesis and overcome plant immunity. The rice blast fungus *Magnaporthe oryzae*, a model for studying fungal-plant interactions, is the first fungal pathogen with all three MAPK pathways functionally characterized (Hamel et al. 2012; Jiang et al. 2018). Therefore, we organized this section based on the *M. oryzae* MAPKs (Fig. 1) and their homologs in *S. cerevisiae* (Table 1). To date, all three MAPK pathways have been characterized in a number of plant pathogenic ascomycetes, including *Alternaria brassicicola*, *Bipolaris sorokiniana, Botrytis cinerea, Cryphonectria parasitica*, *Fusarium graminearum, F. oxysporum,* and *Zymoseptoria tritici* (Leng and Zhong 2015; So and Kim, 2017; Jiang et al. 2018; Francisco et al. 2020). In general, at least two of the MAPK pathways play critical roles in pathogenesis. In some fungi such as *F. graminearum*, all three are important for plant infection (Wang et al. 2011). However, the exact infection-related functions of each MAP kinase pathway may vary significantly among different fungal pathogens.

**Table 1:** Key components of the IG, CWI, and HOG MAPK pathways in *M. oryzae* and *F. graminearum*, and their orthologs in *S. cerevisiae*

	***S. cerevisiae***	***M. oryzae***	***F. graminearum***	Functions
Kss1/Pmk1 IG	Ste2	Ste2^b^	Pre2	GPCR
	Ste3	Ste3^b^	Pre1	GPCR
	Msb2	MoMsb2	FGSG_01127^a^	Mucin
	–	Cbp1	–	GPCR
	–	Pth11	FGSG_08408	GPCR
	–	–	Giv1	GPCR
	Gpa1	MagA	FgGpa1	G-alpha
	Ste4	Mgb1	FgGpb1	G-beta
	Ste18	MGG1	FgGpg1	G-gamma
	Ras2	MoRAS2	FgRAS2	GTPase
	Ste20	Mst20^b^	FgSte20	PAK
	Cla4	Chm1^b^	FgCla4	PAK
	Ste5	–	–	Scaffold
	Ste50^c^	Mst50	FgSte50	Adaptor
	Ste11	Mst11	FgSte11	MEKK
	Ste7	Mst7	FgSte7	MEK
	Fus3/Kss1	Pmk1	Gpmk1 (Fmk1)	MAPK
	Ste12	Mst12	FgSte12	TF
	Mcm1	MoMcm1	FgMcm1	TF
	Sfl1	MoSfl1	FgSfl1	TF
	–	Tpc1	FGSG_08769	TF
**Slt2 CWI**	Wsc1	MGG_04325	FgWsc1	Sensor-transducer
	Wsc2	MGG_02754	FgWsc2/2B	
	Wsc3	MGG_01466	FgWsc3	
	Mid2	MGG_12606	FGSG_08788	Sensor
	Mtl1	–	–	Sensor
	Rho1	MGG_07176	FGSG_04400	Rho
	Pkc1	MoPkc1	FgPkc1	PKC
	Bck1	Mck1	FgBck1	MEKK
	Mkk1/Mkk2	Mkk2	FgMkk1	MEK
	Slt2	Mps1	Mgv1	MAPK
	Swi6	MoSwi6	FgSwi6	TF
	Swi4	MGG_	FGSG_04220	TF
	Rlm1	Mig1	FgRlm1	TF
	Wor1	MoGti1	Fgp1	TF
**Hog1 HOG**	Sln1	MoSln1	FgSln1	Histidine kinase
	Sho1^d^	MoSho1	FgSho1	Osmosensor
	–	MoHik1	FGSG_07118	Histidine kinase
	Ypd1	MoYpd1	FGSG_04363	Phosphotransfer protein
	Ssk1	MoSsk1	FGSG_08948	MEKK
	Ssk2/Ssk22	MoSssk2	FgSsk2	MEKK
	Pbs2	MoPbs2	FgPbs2	MEK
	Hog1	Osm1	FgHog1	MAPK
	Skn7	MoSkn7	FgSkn7	TF
	Atf1	MoAtf1	FgAtf1	TF

- No homolog

^a^Predicted gene number (Homolog are present but not characterized)

^b^Homologs not important for appressorium formation

^c^ Ste50 is functionally related to multiple MAPKs in *S. cerevisiae* and *M. oryzae*

^d^ Sho1 functions mainly in the HOG pathway as an osmosensor in the budding yeast although it ortholog play an important role together with Msb2 to regulate the IG pathway in fungal pathogens

### The Kss1/Pmk1 invasive growth (IG) MAPK pathway

In *S. cerevisiae*, Kss1 and Fus3 are two partially redundant MAPKs that have overlapping functions in pheromone response and filamentation or invasive growth into agar (Morillon et al. 2000). Most filamentous ascomycetes have only one MAPK that is orthologous to Fus3/Kss1 (Table 1). In plant pathogenic fungi, this conserved MAPK cascade is generally required for plant infection and best characterized in *M. oryzae* and the corn smut fungus *Ustilago maydis* (Muller et al. 2003; Li et al. 2012). Pmk1, the first MAPK gene characterized in plant pathogens, is essential for appressorium formations in *M. oryzae*. It also plays a critical role in appressorium penetration and invasive growth (IG) or cell-to-cell spreading *in planta* (Xu and Hamer 1996; Sakulkoo et al. 2018). Pmk1 is activated by its upstream Mst7 MEK and Mst11 MEKK (Fig. 1), but *M. oryzae*, like other fungi, lacks a distinct homolog of yeast scaffold protein Ste5. Instead, Mst50 functions as an adaptor protein that interacts with Mst11 and Mst7 as well as Ras proteins (Zhao and Xu 2007; Zhou et al. 2014). Neither *MST20* nor *CHM1*, two PAK kinases in *M. oryzae*, is required for appressorium formation. *RAS2* is an essential gene and Ras2 likely functions upstream from both the cAMP-PKA and MAPK pathways (Zhou et al. 2012; Qi et al. 2015). In *M. oryzae*, trimeric G-proteins and G protein-coupled receptor (GPCR) Pth11 also are involved in regulating appressorium formation and plant infection, mainly via cAMP signaling (DeZwaan et al. 1999; Nishimura et al. 2003). Msb2 mucin and Sho1 act upstream from the Pmk1 cascade as sensors for plant surface chemicals such as primary alcohols (Liu et al. 2011). For physical signals, a putative chitin-binding protein may play a role in sensing a hydrophobic surface for appressorium formation (Kamakura et al. 2002). MoEnd3 mediated receptor endocytosis for Pth11 and MoSho1 and transduce signals into intercellular compartment in *M. oryzae* (Li et al. 2017a, 2017b). For downstream targets, besides Mst12, Mcm1 and Sfl1 are other two transcription factors that may be regulated by Pmk1 for appressorium penetration and invasive growth (Park et al. 2002; Li et al. 2011; Zhou et al. 2011). *MST12* is essential for pathogenicity and likely regulates septin-mediated cytoskeleton reorganizations in appressoria for penetration. Pmk1 is also essential for hyphopodium formation and root infection as well as the development of appressorium-like structures at hyphal tips (Kong et al. 2013).

In *U. maydis*, a basidiomycete, Kpp2 and Kpp6 are MAPKs with overlapping functions in plant infection and the *kpp2 kpp6* double mutants are nonpathogenic. Kpp6 plays a more important role than Kpp2 in appressorium penetration, but both *kpp2* and *kpp6* deletion mutants are reduced in virulence (Muller et al. 2003; Hu et al. 2007). Interestingly, transcription factor Prf1 appears to function downstream from both the cAMP-PKA and MAPK pathways for regulating plant infection processes (Kaffarnik et al. 2003). In *M. oryzae,* which lacks a Prf1 homolog, Sfl1 also may be coregulated by the Pmk1 MAPK and cAMP signaling pathways (Li et al. 2011; Li et al. 2017a, 2017b). For upstream sensors, Msb2 and Sho1 also are important for appressorium development in *U. maydis* (Lanver et al. 2010). Their homologs in *B. cinerea* and *F. oxysporum* play critical roles in plant infection as well (Leroch et al. 2015; Perez-Nadales and Di Pietro 2015)*.* It is likely that these two sensors are conserved in other fungal pathogens for recognizing plant surface chemical signals to activate the IG MAPK cascade for regulating infection-related development. In fact, in all the appressorium-forming pathogens, including *B. sorokiniana, Colletotrichum gloeosporioides, C. lagenarium*, and *C. heterostrophus*, the IG MAPK pathway is required for appressorium formation (Li et al. 2012; Leng and Zhong 2015; Liang et al. 2019). Furthermore, expression of the *PMK1* orthologs from *C. lagenarium* and *Puccinia striiformis* rescues the defect of *pmk1* in appressorium formation, indicating the well-conserved nature of this MAPK (Jiang et al. 2018).

*PMK1* orthologs also are important for plant infection in various fungal pathogens that do not form appressoria, including vascular wilt pathogens *F. oxysporum* and *V. dahliae,* the wheat scab fungus *F. graminearum*, corn stalk and ear rot pathogen *F. verticillioides*, canker pathogen *C. parasitica*, and biotrophic pathogen *Claviceps purpurea* (Hamel et al. 2012; Li et al. 2012; Jiang et al. 2018). In *Mycosphaerella graminicola* and *Stagonospora nodorum,* the Pmk1 ortholog is important for invasion through stomata and growth in mesophyll tissues (Solomon et al. 2005; Cousin et al. 2006). Genes of diverse functions are found to be regulated by the IG MAPK pathway, such as *PTH11* GPCR, *GAS2/GAS2*, and *MoHOX7* homeobox TF in *M. oryzae*, cell wall degrading enzyme (CWDE) genes in *F. oxysporum, F. graminearum*, and *Valsa mali*, and pheromone precursors in *C. parasitica* (Kim et al. 2009; Li et al. 2012; Wu et al. 2017). Interestingly, this MAPK pathway also regulates the biosynthesis of deoxynivalenol (DON) in *F. graminearum* and fumonisins in *F. verticillioides* (Wang et al. 2011; Zhang et al. 2011). These mycotoxins also are toxic to plant cells and DON is a critical virulence factor in the wheat scab fungus*.*

In summary, the IG MAPK is well conserved for regulating plant penetration and invasive growth in phytopathogenic fungi. Although the exact developmental and infection processes that it regulates vary among different fungi, one common theme is that this MAPK regulates the arrest of germ tube or hyphal tip growth before re-establishing polarized growth for penetrating and invading plant tissues. In addition, this MAPK pathway may regulate the expression of stage-specific genes during disease development, likely in response to plant signals recognized at different infection stages. Besides surface cues, other possible plant signals known to activate the IG MAPK cascade include ethylene in *C. gloeosporioides* (Kim et al. 2000), secreted class III peroxidases in *F. oxysporum* (Turrà et al. 2015), and wheat floral tissue extract in *F. graminearum* (Jiang et al. 2019).

### The Slt2/Mps1 Cell Wall integrity (CWI) pathway

The CWI pathway is required for remodeling of the fungal cell wall during growth, development, and for responding to environmental stimuli. In *S. cerevisiae*, cell wall stresses activate the small G protein Rho1, which then activates the Slt2 MAPK cascade (Table 1) via Pkc1 to regulate gene expression by transcription factors Rlm1 and Swi6 (Jiménez-Gutiérrez et al. 2020). In *M. oryzae*, the *mps1* mutant forms melanized appressoria but is defective in penetration, infectious growth, and sporulation (Xu et al. 1998). Deletion of its upstream MEKK Mck1 results in similar defects in cell wall integrity and plant infection (Jeon et al. 2008). Similar to *mps1* and *bck1* mutants, the *mig1* (MoRlm1) deletion mutant is nonpathogenic and defective in the differentiation and growth of invasive hyphae, likely due to defects in overcoming defense responses (Mehrabi et al. 2008). The *Moswi6* mutant has defects in appressorium turgor generation and forms small specks, but not typical blast lesions, on rice leaves (Qi et al. 2012). Mps1 also controls MoGti1, a transcription factor important for penetration peg formation and expression of several effector genes in *M. oryzae* (Li et al. 2016) (Fig. 1).

The CWI MAPK pathway also has been characterized in a number of plant pathogenic fungi, including *B. cinerea, C. parasitica, C. purpurea, F. graminearum, M. graminicola,* and *Sclerotinia sclerotiorum* (Sanz et al. 2017; Jiang et al. 2018). In general, mutants deleted of the Slt2 ortholog and its upstream MEK or MEKK are significantly reduced in virulence or are non-pathogenic, indicating a conserved role of the CWI MAPK pathway during plant infection. However, infection processes regulated by this pathway differ among fungal pathogens. For example, unlike in *M. oryzae*, the Slt2 ortholog is important for appressorium development in *C. lagenarium* and *C. gloeosporioides* (Kojima et al. 2002; Yong et al., 2013). Whereas the *MgSlt2* mutant is normal in stomata penetration but defective in developing invasive hyphae in *M. graminicola* (Mehrabi et al. 2006), *SMK3* is important for initial infection and sclerotium formation but not for lesion expansion in *S. sclerotiorum* (Bashi et al. 2016).

In *M. oryzae*, the *mps1* mutant has a normal growth rate but produces only limited aerial hyphae. Its orthologs also are dispensable for normal growth rate in *Colletotrichum* species. However, mutants deleted of the Slt2 ortholog have severe growth defects in other fungi, such as *F. graminearum, B. cinerea,* and *C. parasitica* (Hou et al. 2002; Rui and Hahn 2007; So and Kim, 2017). Interestingly, the *Cpslt2* and *Cpbck1* mutants of *C. parasitica* often produce spontaneous suppressors that have a faster growth rate but are still defective in plant infection (So and Kim, 2017), suggesting different roles of the CWI MAPK pathway during vegetative and infectious growth. In *F. graminearum*, deletion of *FgHOG1* partially rescues the growth defect of the *mgv1* mutant but not its defect in pathogenesis (Ren et al. 2019).

### The Hog1/Osm1 high-Osmolarity glycerol (HOG) MAPK pathway

Whereas the other two fungal MAPKs have the TEY dual phosphorylation site, Hog1 and its orthologs have the TGY motif, which is similar to p38 stress activated MAP kinases (SAPKs) in animals. In yeast, the Ssk2/Ssk22-Pbs2-Hog1 MAPK cascade is activated by Sln1 and Sho1 (Table 1), two partially redundant but mechanistically distinct sensors, to mainly regulate responses to hyperosmotic stress (Brewster and Gustin 2014). In *M. oryzae*, the *osm1* deletion mutant is defective in osmoregulation in hyphae but normal in appressorium turgor generation and plant infection (Dixon et al. 1999). Its upstream sensor histidine kinases MoSln1 and MoHik1, phosphotransfer protein MoYpd1p, and response regulator MoSsk1 MEKK (Table 1) also are important for osmoregulation but dispensable for pathogenesis (Jacob et al. 2016) (Fig. 1). However, the Sho1 homolog plays an important role in activating the IG MAPK pathway together with Msb2 for plant infection than for osmoregulation in *M. oryzae* (Liu et al. 2011).

Like in *M. oryzae*, the Hog1/Osm1 ortholog is dispensable for plant infection in several fungal pathogens such as *Cochliobolus orbiculare* and *Bipolaris oryzae* (Moriwaki et al. 2006; Jiang et al. 2018). However, the HOG MAPK pathway is important for plant infection in other plant pathogenic fungi (Jiang et al. 2018). For example, the *hog1* mutant is nonpathogenic and defective in the switch to hyphal growth in *M. graminicola* (Mehrabi et al. 2006). In *B. cinerea*, Sak1 is important for appressorium development and penetration of plant epidermal cells (Liu et al. 2008). In *F. graminearum*, the *Fghog1* mutant is defective in plant infection and DON production (Zheng et al. 2012). In *Ustilaginoidea virens*, UvHog1 regulates the production of secondary metabolites that are toxic to plant cells (Zheng et al. 2016). These observations indicate that the HOG pathway likely plays a species-specific role during plant infection in fungal pathogens. Interestingly, the *C. sativus Cshog1* mutant is normal in root infection but significantly reduced in virulence on barley leaves (Leng and Zhong 2015). In *C. parasitica*, whereas the other two MEK kinase genes are important, the Cpkk3 MEK (CpPbs2) is dispensable for pathogenesis on chestnut (Moretti et al. 2014). However, the *Cpmk1* MAPK (CpHog1) mutant is slightly reduced in virulence in a different *C. parasitica* strain (Park et al. 2004). Therefore, the HOG MAPK pathway may have strain-specific and tissue-specific roles during plant infection as well.

The best characterized downstream target of the HOG MAPK in plant pathogens is the Atf1 (a CREB-like) bZIP transcription factor. In *F. graminearum*, Atf1 interacts with FgOs2 (FgHog1) in the nucleus under osmotic stress, and constitutive expression of *FgATF1* partially complements the defects of *Fgos-2* mutant in osmoregulation and pathogenesis (Van Nguyen et al. 2013). Atf1 orthologs also are important for virulence in *F. verticillioides, M. oryzae, V. dahlia*, and other fungal pathogens (Jiang et al. 2018; Szabó et al. 2020; Tang et al. 2020; Liu et al. 2020). However, in these fungi, *ATF1* orthologs mainly regulate response to oxidative stress instead of hyperosmotic stress. Furthermore, responses to oxidative stress often involve the CWI as well as HOG MAPK pathways as described below.

## MAPK signaling in regulating abiotic stress responses

**Fig. 2:**
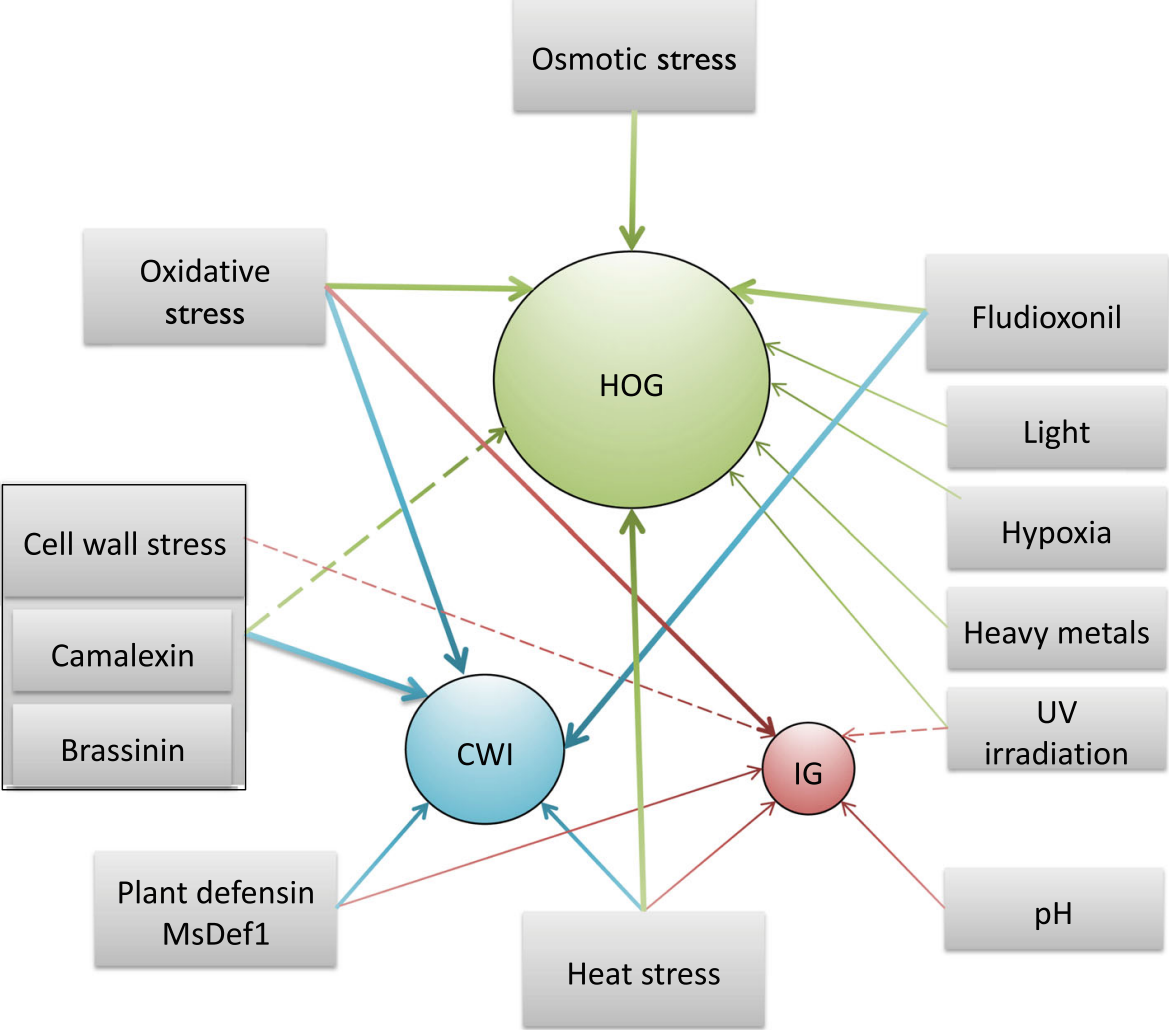
Regulation of responses to abiotic stresses by MAPK Signaling. In general, the HOG pathway plays a major role in regulating responses to osmotic and oxidative stresses in fungi. It is also a major regulator for responses to various environmental stresses, such as UV irradiation, heavy metals, and heat stress in many fungal pathogens. The CWI MAPK pathway mainly regulates responses to cell wall stresses caused by lytic enzymes, cell wall disturbing chemicals, and camalexin and brassinin phytoalexins. In some plant pathogenic fungi, the CWI and HOG MAPKs crosstalk to regulate responses to cell wall and oxidative stresses. In comparison with the other two MAPKs, the IG MAPK generally plays less important roles in regulating stress responses in fungal pathogens although it may be involved in the co-regulation of responses to heat, pH, and other environmental stresses together with the HOG or CWI pathway. Nevertheless, in some fungi, the IG and CWI MAPKs regulate melanin synthesis and melanization of cell wall confer resistance to various stresses

In *S. cerevisiae*, Slt2 and Hog1 mainly regulate responses to cell wall and hyperosmotic stresses, respectively, although these MAPKs, particularly Hog1, also are involved in other stress responses (Ikner and Shiozaki 2005; Serrano et al. 2006; Brewster and Gustin 2014). In comparison, MAPK pathways in phytopathogenic fungi generally play more important roles in response to various environmental stresses (Fig. 2), including antifungal chemicals, reactive oxidative species (ROS), elevated temperatures, UV irradiation, and plant defense compounds (Lee et al. 2017; Dunayevich et al. 2018; Yang et al. 2020).

### Cell wall stress

The fungal cell wall is not only important for maintaining morphology but also for protecting against environmental stresses. Like in *S. cerevisiae*, the CWI MAPK pathway plays a critical role in regulating responses to cell wall stress in plant pathogenic fungi, and mutants deleted of the Slt2/Mps1 ortholog have increased sensitivities to cell wall lytic enzymes and cell wall stressors such as Congo Red (CR) or Calcofluor White (CFW) (Hamel et al. 2012; Jiang et al. 2018). Autolysis of aerial hyphae is observed in old cultures of the *mps1* and *bck1* mutants in *M. oryzae* and *Aspergillus flavus* (Xu et al. 1998; Jeon et al. 2008; Zhang et al. 2020; Feng et al. 2021). In *S. cerevisiae*, cell stressors, such as CR and Caspofungin, are sensed by the Mid2 and Wsc1 sensors (Jin et al. 2013). Similar sensors appear to be involved in sensing these cell wall stressors in fungal pathogens, such as *U. maydis* and *F. graminearum* (Carbó and Pérez-Martín 2010; Xu et al. 2019). In yeast, cell wall damage caused by β-1,3-glucanase and protease activities is sensed by Sho1 and mucin Hkr1 (not Msb2) to activate Hog1, which in turn activates Slt2 (Rodríguez-Peña et al. 2013). Although whether mediated by the CWI MAPK or not is not clear, the involvement of the HOG pathway in responding to cell wall stress also has been observed in plant pathogenic fungi such as *F. graminearum* and *S. sclerotiorum* (Zheng et al. 2012; Duan et al. 2013). In *A. brassicicola*, both CWI and HOG pathways are involved in response to cell wall stress caused by camalexin and brassinin, two indolic phytoalexins produced by *Brassica* species (Joubert et al. 2011).

In some plant pathogenic fungi, the IG MAPK pathway also plays a role in cell wall stress responses. However, its importance and regulatory functions vary among different fungal pathogens. For example, deletion of ChMK1 results in hypersensitivity to CFW and CR in *C. higginsianum* (Wei et al. 2016), but the *Cfpmk1* mutant has increased tolerance against CR and SDS in *C. fructicola* (Liang et al. 2019). Whereas the *CcPmk1* mutant is hypersensitive to cell wall stress and cell wall lytic enzymes in *Cytospora chrysosperma* (Yu et al. 2019)*,* the *chk1* mutant has the hyphal autolysis and aerial hyphal growth defects in *C. heterostrophus* (Lev et al. 1999). In *F. graminearum*, both Gpmk1 and Mgv1 MAPKs are involved in regulating basal resistance to plant defensin MsDef1 but not MtDef4 (Ramamoorthy et al. 2007). In *M. oryzae*, deletion of a Pmk1-interacting gene *PIC5* results in increased sensitivity to cell wall lytic enzyme (Zhang et al. 2011). In *F. oxysporum*, deletion of *FMK1* in the wild type has no obvious effect on sensitivity to cell wall stress but increases the sensitivity of the *mpk1* (Slt2) mutant (Segorbe et al. 2017), suggesting a minor role of the IG MAPK in cell wall integrity.

### Osmotic stress

Plant pathogenic fungi may face hyperosmotic stress during infection of tissues with high sugar contents and survival in desiccated plant tissues. The HOG pathway is conserved in fungal pathogens for regulating adaptive responses to hyperosmotic stress, including the synthesis and retention of compatible osmolytes such as glycerol, arabitol, and sorbitol (Dixon et al. 1999; Zheng et al. 2016; Li et al. 2020). Deletion of *HOG1* orthologs results in increased sensitivity to hyperosmotic stress in all the plant pathogenic fungi studied. Like in yeast, Hog1 orthologs are rapidly phosphorylated in response to hyperosmotic stress in fungal pathogens such as *C. heterostrophus* (Yoshimi et al. 2005). For the upstream histidine kinases of the HOG pathway in *M. oryzae*, the *Mosln1* mutant is more susceptible to salt stress, but the *Mohik1* mutant is more sensitive to sugar stress (Jacob et al. 2014). Downstream transcription factors of the HOG pathway that have been shown to regulate osmoregulation in fungal pathogens include Atf1 and Skn7 (Zheng et al. 2016; Tang et al. 2020). However, these transcription factors have species-specific functions in osmoregulation. For example, Atf1 is more important in *F. graminearum* but Skn7 is more important in *B. cinerea* for regulating responses to osmotic stress (Jiang et al. 2015; Viefhues et al. 2015; Yang et al. 2015).

Interestingly, knocking down the expression of *PiHOG1* in *Piriformospora indica*, an endophyte of rice roots, results in an increased sensitivity to osmotic stress not only in the fungus but also in rice plants colonized by the knocked down strain (Jogawat et al. 2016). The accumulation of compatible osmolyte proline is reduced in rice roots inoculated with this strain, suggesting that the endophytic fungus may confer osmotic stress tolerance to the host plant by upregulating proline production via the HOG MAPK. Although likely irrelevant to plant pathogens, it is worth noting that the HOG pathway also regulates response to hypoosmotic stress in the halophilic fungus *Wallemia ichthyophaga*, which has two functional Hog1 MAPKs (Konte and Plemenitas 2013).

### Oxidative stress

Oxidative stress can be caused by ROS generated intracellularly or exposure to oxidants from the environment or host plants. In many plant pathogenic fungi, the HOG pathway plays a critical role in oxidative stress response. Mutants deleted of the Hog1 MAPK or other key components of this pathway have increased sensitivity to oxidative stress, and some are defective in plant infection as described above. The Atf1 ortholog is one major TF functioning downstream from the HOG MAPK to regulate genes important for oxidative responses in fungal pathogens (Guo et al. 2011; Tang et al. 2020). Homologs of the response regulator Skn7, another component of the HOG pathway, also is important for oxidative stress response in fungal pathogens such as *A. alternata* (Chen et al. 2012), likely by regulating the expression of oxidative stress-induced genes, including those encoding catalases and superoxide dismutase (Fassler and West 2011). Nevertheless, in some fungi such as *M. oryzae*, the Skn7 ortholog is dispensable for response to oxidative stress and pathogenesis (Motoyama et al. 2008).

Mutants deleted of key components of the CWI MAPK pathway also have increased sensitivities to oxidative stress in some fungal pathogens such as *B. cinerea* (Yin et al. 2018) and *F. verticillioides* (Zhang et al. 2015). However, it is not clear whether this MAPK regulates stress response genes directly or by crosstalk with the HOG pathway in these fungi. In yeast, under low or moderate oxidative stress conditions, the highly O-mannosylated Mtl1 protein acts together with Wsc1 or its paralog Mid2 as the sensors of the CWI pathway to activate Slt2, which results in the translocation and degradation of cyclin C, a negative regulator of genes involved in stress responses (Vilella et al. 2005; Jin et al. 2013). Homologs of Mtl1 and Mid2 are present in the genomes of fungal pathogens but none of them have been functionally characterized.

AP1 is another b-ZIP transcription factor (conserved from yeast to human) that acts as a redox-responsive regulator for regulating oxidative stress-related genes. The C-terminal domain of Yap1 contains cysteine residues that can form an intramolecular disulfide bridge under oxidizing conditions, which enable its localization to the nucleus for activating its target genes. Although AP1 orthologs are involved in oxidative stress responses in general, their importance for plant infection vary among fungal pathogens. For example, the *ChAP1* deletion mutant of *C. heterostrophus* is increased in sensitivity to H_2_O_2_ but has no obvious defect in virulence (Lev et al. 2005). However, mutants deleted of the AP1 ortholog are hypersensitive to oxidative stress and defective in plant infection in *A. alternata, C. gloeosporioides,* and *M. oryzae* (Lin et al. 2010; Guo et al. 2011; Sun et al. 2016)*.* Nevertheless, the functional relationship between AP1 and MAPKs remains to be clarified in these pathogens. In the budding or fission yeast, Yap1 is a redox-sensitive transcription factor that is not known to be a direct substrate of Hog1/Sty1 MAPK, suggesting an indirect relationship. In *M. oryzae*, Mst7 interacts with thioredoxin Trx2, which is a target of MoAP1 and important for response to H_2_O_2_ or diamide (Zhang et al. 2016; Wang et al. 2017), suggesting a possible indirect relationship between of Pmk1 and MoAP1. The Tpc1 transcription factor that regulates NOXD expression in *M. oryzae* interacts with Mst12 (Galhano et al. 2017), further implicating the involvement of the IG MAPK pathway in responses to oxidative stress.

### Antifungal chemicals

Treatments with fungicides or antifungals with different modes of actions likely cause target-specific stresses in fungi, which may trigger MAPK-mediated responses. One well-characterized example is the roles of multiple MAPKs in responding to cell wall stress caused by Caspofungin, CR, and CFW described above (2.1). Another example is the over-stimulation of the HOG pathway by phenylpyrrole fungicides fludioxonil and fenpiclonil. First discovered in *Neurospora crassa*, treatments with fludioxonil results in the accumulation of intracellular glycerol and cell burst, and null mutations in *OS-2* (*HOG1*) confer resistance against these fungicides (Zhang et al. 2002). Fludioxonil also stimulates cell burst and Hog1 overactivation in *C. lagenarium* (Kojima et al. 2004), and mutants deleted of the Hog1 ortholog are resistant to fludioxonil in yeast and other plant pathogenic fungi (Jiang et al. 2018). Deletion of the HOG1 ortholog or upstream components of the HOG pathway in fungal pathogens, including homologs of yeast *PBS2*, *SSK2/SSK2*, *SLN1*, *YPD1*, and *HIK1* also confer resistance or tolerance to fludioxonil. However, none of these HOG components are the direct target of fludioxonil. A recent study suggested that fludioxonil may target and inhibit triosephosphate isomerase, resulting in elevated cytosolic methyglyoxal, which in turn changes a Sln1-like group III histidine kinase into a phosphatase to constitutively activate the downstream Hog1 MAPK cascade (Brandhorst et al. 2019). Remarkably, fludioxonil and fenpiclonil have been applied to control foliar pathogens for over 30 years, but field isolates with complete resistance against these phenylpyrrole fungicides have not emerged and spread widely in crop fields (Kilani and Fillinger 2016), which may be related to the defects of HOG mutants in stress response and survival in nature.

Interestingly, the *os-2*, *os-5* (*PBS2*), and *ssk22* deletion mutants in *N. crassa* are also resistant to dicarboximide fungicides vinclozolin and iprodione (Zhang et al. 2002; Fujimura et al. 2003). Resistance against dicarboximide fungicides also has been observed in mutants defective in the HOG pathway in plant pathogens (Jiang et al. 2018). For example, in *Alternaria alternata*, the *hog1*, *ssk1*, *skn7*, and *hsk1* deletion mutants all express increased tolerance at various degrees against fludioxonil and vinclozolin, with the *hsk1* mutant having the highest level of tolerance (Yu et al. 2016). However, although stimulation of glycerol accumulation has been reported (Ochiai et al. 2002), the actual target of dicarboximide fungicides is not certain. In some fungi, mutants in the cAMP-PKA pathway and PKC also confer resistance against dicarboximide fungicides (Ramesh et al. 2001; Mehrabi et al. 2006). In addition, the *chk1* and *mps1* mutants of *C. heterostrophus* are slightly increased in tolerance, but the *F. graminearum mgv1* mutant is increased in sensitivity to fludioxonil (Degani 2015; Ren et al. 2019), suggesting that the other two MAPKs may play minor roles in fludioxonil tolerance, likely by crosstalk with the HOG pathway.

### Heat stress (HS)

Fungal pathogens must tolerate elevated temperatures in the field. Although the functions of MAPK pathways in heat stress (HS) responses, particularly the CWI pathway, are well characterized in the budding yeast, such as the activation of Slt2 via Cbk1 and Bck2, and Hog1 phosphorylation due to glycerol loss at elevated temperatures (Kuravi et al. 2011; Dunayevich et al. 2018), there are only limited studies on the regulation of heat tolerance by MAPKs in plant pathogenic fungi. The *mgv1* mutant of *F. graminearum* has increased sensitivity to elevated temperatures, which is partially suppressed by deletion of FgHog1 (Ren et al. 2019). Like other organisms, fungi produce heat shock proteins (HSPs) and chaperones to protect proteins from aggregation and degradation. In yeast, Sfl1 is a heat shock factor-like transcriptional regulator that controls the expression of Hsp30 at 42 °C (Galeote et al. 2007). In *M. oryzae*, MoSfl1 functions downstream from both the cAMP signaling and Pmk1 MAPK pathways and the *Mosfl1* deletion mutant has increased sensitivity to elevated temperatures (Li et al. 2011; Li et al., 2017).

The MoSsb1 HSP70-like protein interacts with MoMkk1, the MEK for Mps1, and regulates its expression. MoSsb1 forms protein complexes with MoSsz1 (another member of HSP70) and MoZuo1 (a HSP40 protein) that are important for tolerance to temperature stress in *M. oryzae* (Yang et al. 2018). HSP90, one of the most ubiquitous chaperones, facilitates the activation of Slt2 in response to heat stress, and its client proteins that include Hog1 in *S. cerevisiae* and *Candida albicans* (Leach et al. 2012). In plant pathogens, HSP90 likely has similar functions in response to heat and other stresses via its association with MAPKs. Interestingly, some plant pathogens produce resorcyclic acid lactones such as zearalenone, which may be inhibitory to plant and fungal HSP90 proteins during their interactions with host plant (pathogenesis) or other fungi (competition).

### Responses to other environmental factors

Although there are only limited studies, fungal MAPKs also have been implicated in responding to other environmental stresses. In *M. oryzae*, vegetative growth of the *Mohik5* and *Mohik9* mutants is sensitive to hypoxia-inducing NaNO_2_ and treatment with NaNO_2_ resulted in Osm1 phosphorylation, suggesting a role for the HOG pathway in response to hypoxia (Jacob et al. 2014). In *Trichoderma atroviride*, silencing of *ThHOG1* (*TMK3*) results in a minor increase in sensitivity to 40 mM CuSO_4_ (heavy metal stress) that stimulates ThHog1 phosphorylation (Delgado-Jarana et al. 2006). The HOG pathway is also involved in regulating DNA repair in *T. atroviride* because the *tmk3* and *pbs2* mutants are highly sensitive to UV irradiation when incubated in dark (Esquivel-Naranjo et al. 2016). Furthermore, light stimulates Tmk3 phosphorylation, and deletion of *TMK3* affects the expression of genes induced by blue light (Esquivel-Naranjo et al. 2016). The HOG pathway is known to be involved in light response and circadian rhythm in the model filamentous ascomycetes *N. crassa* and *Aspergillus nidulans* (Bennett et al. 2013; Yu et al. 2021).

In *F. oxysporum*, alkaline pH induces the rapid phosphorylation of Fmk1 MAPK, whereas shifting to acidic pH (5.0) results in its dephosphorylation (Masachis et al. 2016). Because rapid alkalization or acidification of infected tissues may be parts of plant defense responses, it is possible that fungal pathogens use changes in pH to trigger the expression of certain effectors or virulence factors via the IG MAPK pathway. The biosynthesis of melanin, an excellent photoprotectant, is also known to be regulated by the IG MAPK pathway in plant pathogenic fungi, such as *C. gloeosporioides*, *C. heterostrophus*, and *V. dahliae* (Lev et al. 1999; Rauyaree et al. 2005; He et al. 2017). Melanization of the cell wall provides protection against various stresses, including ionizing and UV irradiation, host defensive compounds, elevated ROS, and fungivores. In fact, many fungi that live in extreme habitats such as extreme cold are melanized (Gessler et al. 2014). Regulation of melanin synthesis by the IG MAPK pathway suggests its indirect involvement in response to various environmental stresses, likely together with the HOG and/or CWI MAPKs. In *C. heterostrophus*, both Chk1 and Mps1 MAPKs are involved in the regulation of *CMR1* and melanin synthesis (Eliahu et al. 2007).

## Roles of MAPK signaling in biotic stress responses

**Fig. 3:**
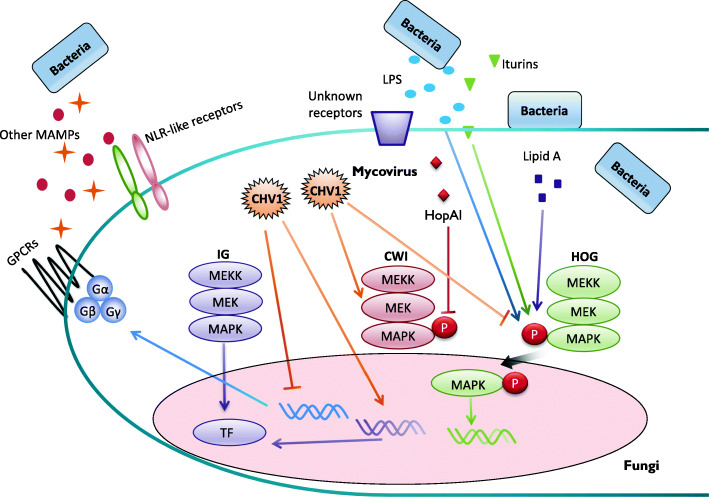
Roles of MAPK signaling in fungal interactions with viruses and bacteria. Although with only limited studies, the HOG and CWI pathways have been implicated in bacteria-fungal interactions (BFIs). For examples, iturins, a special class of pore-forming lipopeptides secreted by *B. amyloliquefaciens*, and *E. coli* lipopolysaccharide (LPS) or endotoxin lipid A stimulate the phosphorylation of Hog1. Activated Hog1 MAPK localizes to the nucleus and regulates the expression of *GPD1* and other downstream targets. Whereas the recognition of MAMPs (microbe-associated molecular pattern) such as LPS by GPCR and NLR-like receptors in fungal pathogens may lead to the activation of MAPK pathways, bacteria may secrete effector proteins to suppress the activation of MAPKs involved in BFIs. In *M. oryzae*, expression of the bacterial effector HopAI significantly reduces the phosphorylation of Mps1 and therefore inactivates the CWI signaling. Similarly, fungal pathogens likely utilize MAPKs to regulate defense against mycoviruses but mycovirus infection may directly or indirectly interfere with MAPK signaling in fungal pathogens. In *C. parasitica*, CHV1 infection reduces the expression of trimeric G-proteins and downstream Ste12 transcription factor of the IG MAPK pathway. CHV1 infection also affects the phosphorylation of the MEK (positively) of the CWI pathway and Hog1 MAPK (negatively) of the HOG pathway

Besides infection of their hosts, plant pathogenic fungi also have to compete with other microbes in the environment or on plant surface for survival and propagation. In comparison with their roles in pathogenesis and response to abiotic stresses, functions of MAPKs in the interactions of fungal pathogens with mycoviruses, bacteria, and other fungi are under-investigated (Fig. 3).

### Interactions with bacteria

Fungal and bacteria can form a range of physical associations, and bacteria-fungal interactions (BFI) can affect the ecosystems in which they coexist or their associations with host plants or animals (Deveau et al. 2018). Whereas ‘fungiphile’ bacteria are preferentially associated with fungi and mainly live in the ‘hyphosphere’ habitat surrounding hyphae, fungal endobacteria live inside hyphal cells as symbionts. Some bacteria are parasites that degrade fungal cells but others simply use hyphae for bacterial transport, such as the utilization of *F. oxysporum* hyphae by *Rahnella aquatilis* (Palmieri et al. 2020). In these diverse and dynamic physical interactions, both bacteria and fungi are active in signaling and signal recognition for responding appropriately to their partners. Fungal quorum sensing molecules (such as farnesol and tyrosol) and bacterium-secreted metabolites (such as quinolones and homoserine lactones) have been implicated in inter-kindom signaling (Chatterjee et al. 2020; Sharma et al. 2020). However, the roles of fungal MAP kinase pathways in the establishment of BFI or BFI networks have not been well studied. In *S. cerevisiae*, treatments with *Escherichia coli* lipopolysaccharide (LPS) and endotoxically active lipid A stimulate the phosphorylation of Hog1 and its translocation to the nucleus, as well as the expression of its downstream target *GPD1* (Marques et al. 2006). In *V. dahliae*, treatment with iturins, pore-forming lipopeptides, produced by *Bacillus amyloliquefaciens*, activates Hog1 and causes cell wall integrity defects (Han et al. 2015). During symbiotic establishment with *Mycetohabitans* endobacteria, several components of the HOG pathway and its downstream targets were upregulated in *Rhizopus microsporus* (Lastovetsky et al. 2016). These observations indicate a role of the HOG MAPK pathway in fungal-bacteria interactions. Furthermore, the TOR kinase targeted by rifamycin functions upstream from MAP kinase cascades in *S. cerevisiae* and filamentous fungi (Loewith and Hall 2011; Inoue and Nomura 2018), suggesting indirect inhibition of fungal MAPKs by antibiotics produced by bacteria.

Recognition of microbe-associated molecular patterns (MAMPs) by Nod-like immune receptors (NLRs) leads to the activation of downstream MAPKs for regulating defense responses in plants and animals. Secreted effectors of some bacterial pathogens are known to interfere with host MAPK signaling (Shan et al. 2007; Krachler et al. 2011). NLR-like receptor genes are widely distributed in filamentous fungi, and some of them may be involved in MAMP recognition (Uehling et al. 2017) to activate fungal MAPKs for defense response against bacteria. On the other hand, fungal MAPKs may be targeted by bacterial effectors for inhibition. Consistent with this hypothesis, expression of the *Pseudomonas syringae* effector HopAI, a MAPK inhibitor, significantly reduces the phosphorylation level of Mps1 and Mgv1 MAPKs in *M. oryzae* and *F. graminearum* (Zhang et al. 2017). Along the same line, some of the bacterial species used for biocontrol against fungal pathogens may target fungal MAPKs for interfering with their pathogenesis-related functions. To test these hypotheses and better understand the roles of fungal MAPKs in BFI, it will be helpful to have a model plant pathogenic fungus for studying fungal-bacterial interactions during plant infection.

### Fungal-mycovirus interactions

Like plants and animals, fungi are also susceptible to infections by mycoviruses. The best characterized mycovirus is Cryphonectria hypovirus 1 (CHV1) that causes hypovirulence and defects in sexual/asexual development in *C. parasitica*. CHV1 infection interferes with the expression levels of the trimeric G-proteins that function upstream from both cAMP signaling and MAPK pathways (Dawe and Nuss 2013). Although the exact mechanisms are not clear, all three MAPKs in *C. parasitica* are involved in its interactions with CHV1. The first MAPK found to be affected by mycovirus infection is CpMK1 (Hog1). The phosphorylation of CpMK1 under hyperosmotic conditions is reduced in the virus-infected hypovirulent strain, which has increased osmotic sensitivity (Park et al. 2004), indicating the suppression of CpMK1 activation by CHV1.

Unlike CpMK1, the level of CpMK2 (Pmk1) phosphorylation is not affected but its downstream TF CpSte12 is down-regulated by CHV1 infection (Choi et al. 2005; Deng et al. 2007). Furthermore, only the *CpKK2* (CpSte7) deletion mutant, not mutants deleted of the two other MEK genes, could not be infected with CHV1 virus or transformed with infectious CHV1 cDNA via protoplasts (Turina et al. 2016), suggesting a role of this MAPK pathway in mycovirus infection and replication. In *C. parasitica*, *CpPK1*, a Cot-1 homolog, is transcriptionally upregulated by CHV1 and overexpressing CpPK1 reproduces some of the viral symptoms (Kim et al. 2004). CpPK1 may be functionally related to the IG MAPK pathway because spontaneous suppressor mutations in this pathway can partially rescue the *cot-1* mutant, and the scaffold protein Hym1 for the Cot-1 NDR kinase complex is essential for the activation of this MAPK in *N. crassa* (Maerz et al. 2008; Dettmann et al. 2012).

For the CWI pathway, CHV1 partially represses the dephosphorylation of CpKK1 MEK, resulting in its elevated phosphorylation and likely the hyperactivation of its downstream MAPK (Turina et al. 2016). Furthermore, studies in other fungi such as *F. graminearum* and *N. crassa* have showed that the CWI MAPK is essential for hyphal fusion (Hou et al. 2002; Fischer and Glass, 2019). Because cell death triggered by fusion between vegetative incompatible strains protects against the spreading of mycoviruses in fungal hyphae (Dawe and Nuss 2013), the CWI MAPK pathway likely plays an indirect but important role in defense against mycoviruses in plant pathogenic fungi in general.

### Fungal-fungal interactions

Many fungi also co-exist or compete against each other in nature. Whereas some interactions are mediated via antagonistic/inhibitory compounds or metabolites released into the environment, some involve direct hyphal-hyphal contacts, leading to hyphal fusion and mycoparasitism or heterokaryosis. Although signaling compounds and their receptors remain to be identified, two MAP kinase pathways and the Striatin-Interacting protein Phosphatase And Kinase (STRIPAK) complex have been implicated in regulating chemotropism and hyphal fusion, an integrated process of colonial growth in the saprophytic model fungus *N. crassa* and *Sordaria macrospora* (Reschka et al. 2018; Fischer and Glass, 2019). In *N. crassa*, the Soft protein functions as a scaffold for the upstream components of the CWI MAPK pathway and the Soft complexes undergo oscillations of assembly and disassembly (4-min intervals) at hyphal tips during chemotropic interactions. Ham-5 functions as the scaffold protein for the Mak-2 (Kss1) cascade, and the Ham-5 complexes mirror the Soft complexes in 4-min interval oscillations of assembly and disassembly at hyphal tips in interacting hyphae. Mirroring oscillations of the Soft and Ham-5 complexes may be related to their functions in signal secretion and reception, respectively (Goryachev et al. 2012). The STRIPAK complex (Kück et al. 2016) also localizes to the hyphal tip and cross-talks with Mak1 and Mak-2 pathways to regulate hyphal fusion. Besides their physical associations detected by mass spectrometry analysis, key components of the Soft, Ham-5, and STRIPAK complexes are required for full phosphorylation of Mak-1 and Mak-2 MAPK (Dettmann et al. 2014; Fischer and Glass, 2019). Furthermore, PP-1 (Ste12) of the Mak-2 pathway directly activates the expression of Adv-1 that functions downstream from Mak-1 for regulating genes important for hyphal fusion (Fischer et al. 2018). However, the functions of these three complexes in hyphal fusion may be not well conserved in fungal pathogens. In *F. graminearum*, Mgv1 MAPK is essential for hyphal fusion and heterokaryon formation (Hou et al. 2002) but hyphal fusion still occurs in the *Fgso* (*Fgsoft*) and *Gpmk1* deletion mutants (Zheng et al. 2013).

Hyphal fusion can occur between hyphae of the same strain, different strains of the same species, or different species. In *N. crassa*, the *doc* genes regulate non-self recognition before hyphal fusion (Heller et al. 2016), but the Het genes control heterokaryon incompatibility after hyphal fusion (Glass and Kaneko 2003). Whereas fusion between incompatible strains leads to cell death in heterokaryotic cells, heterokaryons are formed between compatible strains, which may lead to parasexual reproduction and somatic recombination to increase genetic variation in asexual fungal pathogens (Clutterbuck 1996; Daskalov et al. 2017). Although studies are lacking, MAPKs may also play regulatory roles in parasexual reproduction processes after hyphal fusion and heterokaryon formation.

Mycoparasitism also involves recognition and hyphal attachment but results in the killing and degradation of host hyphae by the mycoparasite after hyphal fusion or penetration. Mycoparasitic fungi are often facultative parasites that switch from saprophytic to parasitic growth when triggered by host-derived signals such as oligopeptides or oligochitosaccharides and secondary metabolites (Druzhinina et al. 2011; Holzlechner et al. 2016). The best characterized mycoparasitic fungi are *Trichoderma* species, such as *T. atroviride* and *T. virens* that are used for biocontrol of fungal diseases. However, biocontrol involves interactions with the host plant, and some *Trichoderma* species can stimulate plant immune responses. Therefore, whereas the roles of MAPKs in hyphal-hyphal interactions may be conserved during mycoparasitism, their functions in biocontrol may involve species- or strain-specific stimulation of plant immunity.

All three MAPKs named as Tmk1/TmkA (Kss1), Tmk2/TmkB (Slt2), and Tmk3/TmkC (Hog1) have been characterized in Trichoderma species. In *T. virens,* a gliovirin producer, the *tmkA* and *tmkB* deletion mutants both are defective in vegetative growth and reduced in mycoparasitism against *S. rolfsii* but normal against *Rhizoctonia solani*, indicating overlapping functions of these two MAPKs (Mukherjee et al. 2003; Mukherjee et al. 2012; Kück et al. 2016). Whereas TmkB is important for cell wall integrity, Tmk1 regulates mycoparasitism-relatedt genes, showing their distinct functions. In *T. atroviride*, the *tmk1* deletion mutant is reduced in mycoparasitic ability to overgrow host fungi but increased in antifungal activities and ability to protect against *R. solani* infection in bean (Reithner et al. 2007), and TaSte12 is involved in regulating hyphal interactions (coiling) and mycoparasitic activity (Gruber and Zeilinger 2014). These results suggest that the Tmk1/TmkA MAPK has a conserved role in mycoparasitism by regulating the expression of mycoparasitism-related genes (such as chitinase genes and genes responsible for synthesis of antifungal metabolites) and mycoparasitic hyphal interactions. Consistent with these observations, deletion of the *TMK1* ortholog in *Clonostachys chloroleuca*, another mycoparasitic fungus, also affects the expression of cell wall lytic enzymes, mycoparasitic ability, and biocontrol (Sun et al. 2020). However, deletion of this MAPK gene in a gliovirin-deficient mutant of *T. virens* increases mycoparasitic activity against *R. solani* (Mendoza-Mendoza et al. 2003), which is contradictory to the regulation of mycoparasitic hyphal-hyphal interactions by this MAPK pathway.

As expected, Tmk3/ThHog1 regulates responses to hyperosmotic and oxidative stresses in *T. atroviride* and *T. harzianum* (Delgado-Jarana et al. 2006; Esquivel-Naranjo et al. 2016). In *T. harzianum*, deletion of *ThHOG1* reduces its antagonistic activity against host fungi *Phoma betae* and *C. acutatum*. ThHog1 likely plays a role in mycoparasitism by regulating responses to toxic compounds produced by host fungi. Although studies are lacking, the host fungi may also use Hog1 or other MAPKs to regulate defense or antagonistic responses against mycoparasitic fungi or other predators. Interestingly, Tmk3 is involved in the repression of subsets of secondary metabolism (SM) genes that are stimulated by mechanical wounding but suppressed by Drosophila larvae (Atriztán-Hernández et al. 2019). In comparison with the wild type, Drosophila larvae prefer feeding on hyphae of the *tmk3* mutant but have a higher mortality rate, suggesting a role of this MAPK in interactions with fungivorous insects.

## Perspectives

In phytopathogenic fungi, the well-conserved IG, CWI, and HOG MAPKs have both conserved and species-specific functions in regulating plant infection, stress responses, growth, and sexual or asexual development (Hamel et al. 2012; Jiang et al. 2018). However, it is worth mentioning that, to date, most of the MAPK studies in plant pathogenic fungi deal with ascomycetous pathogens. Based on studies in *U. maydis*, the components and functions of MAPK pathways in basidiomycetes are likely different from those in phytopathogenic ascomycetes. Also, most of these MAPK studies in fungal pathogens are based on targeted gene knockout mutants. Proteomics studies in yeast have shown that MAPKs often are hubs in the protein-protein networks (Chen and Snyder 2010). Therefore, some of the phenotypes observed with mutants disrupted in MAPK signaling may be due to indirect effects via protein-protein interaction networks. It will be important to determine the effects of transient inhibition with ATP analog PP-1 on MAPKs with appropriate mutants at the ATP binding site (Sakulkoo et al. 2018) for comparisons.

For all the known upstream sensors or receptors of yeast MAPK pathways, their homologs are present in fungal pathogens, including GPCRs, Sho1, Msb1, Wsc1–3, Mst11, Mid2, and Sln1 (Jendretzki et al. 2011; Alvaro and Thorner 2016; Vázquez-Ibarra et al. 2020). However, in comparison with other sensors, GPCRs are significantly expanded in fungal pathogens. In the budding yeast, three typical GPCR genes encode two pheromone receptors and one glucose sensor. In *M. oryzae*, there are over 40 putative GPCRs, and at least one of them with the CFEM domain, *PTH11*, has been convincingly shown to be important for appressorium formation and virulence (Kulkarni et al. 2005). *F. graminearum* has over 100 putative GPCRs, including 12 CFEM GPCRs and a subfamily of infection-related or specific GPCRs (Jiang et al. 2019). These GPCRs may be involved in sensing different host and environmental signals to activate downstream MAPKs. Some of them may be involved in sensing their own signals (quorum sensing) or ligands from other microbes during fungal-fungal or fungal-bacterial interactions.

Unlike their roles in pathogenesis or mycoparasitism, the roles of fungal MAPK pathways in defensive interactions against viruses, bacteria, and other fungi have not been well studied. Nevertheless, several publications have shown that microbes have the capacity to activate fungal MAPK signaling, possibly by the recognition of microbial signals/ MAMPs by membrane receptors. The presence of putative NLRs in fungal pathogens suggest that MAPK signaling may function downstream from some of those NLRs involved in MAMP recognition. For genes regulated by MAPKs for defense responses, there are only very limited studies. Fungi are known to secrete enzymes, toxins, and other secondary metabolites to compete with other microbes in the environment or on the plant surface. Genes encoding defense-related enzymes or proteins may be regulated by multiple MAPKs, and the activation of multiple MAPK pathways can alter the composition of the surrounding microbiota. Therefore, to better understand the role of fungal MAPKs in biotic interactions, the single, double, or triple MAPK mutants should be comparatively studied for changes in fungal-bacterial or fungal-fungal interactions.

Another important area is to further characterize the functional relationships among these MAPK pathways. A considerable amount of evidence shows the crosstalk between HOG and CWI, CWI and IG, or IG and HOG pathways in fungal pathogens in responses to biotic and abiotic stresses. Nevertheless, fungal pathogens must utilize all these signaling pathways to coordinately regulate responses to a variety of stresses encountered during plant infection or survival in nature. Therefore, it will be helpful to systematically characterize the functional relationships among all three MAPK cascades in responses to different stresses or during different infection and developmental stages. These MAPKs may share some common downstream targets and upstream components or regulators/sensors. Furthermore, all three MAPKs are likely involved in crosstalk with other signaling pathways. For example, the IG MAPK is known to interact with the cAMP-PKA pathways in regulating infection structure formation and invasive growth in *M. oryzae* and other fungi (Jiang et al. 2018). Crosstalk between calcium signaling and CWI and other MAPKs also occur in plant pathogens (Wurzinger et al. 2011). It will be important to use transcriptomics and proteomics approaches to systematically characterize the crosstalk and functional relationships among these well-conserved signal transduction pathways.

## Data Availability

Not applicable.
